# The Influence of COD Fraction Forms and Molecules Size on Hydrolysis Process Developed by Comparative OUR Studies in Activated Sludge Modelling

**DOI:** 10.3390/molecules25040929

**Published:** 2020-02-19

**Authors:** Jakub Drewnowski, Bartosz Szeląg, Li Xie, Xi Lu, Mahesh Ganesapillai, Chinmoy Kanti Deb, Joanna Szulżyk-Cieplak, Grzegorz Łagód

**Affiliations:** 1 Faculty of Civil and Environmental Engineering, Gdansk University of Technology, Narutowicza 11/12, 80-233 Gdansk, Poland; 2 Faculty of Environmental Engineering, Kielce University of Technology, Av. Tysiąclecia Państwa Polskiego 7, 25-314 Kielce, Poland; bszelag@tu.kielce.pl; 3 Faculty of Civil and Environmental Engineering, Tongji University, Siping Road 1239, Yangpu District, Shanghai 200092, China; sally.xieli@tongji.edu.cn (L.X.); luxi953@outlook.com (X.L.); 4 Mass Transfer Group, School of Chemical Engineering, Vellore Institute of Technology, Gorbachev Road, Vellore, Tamil Nadu 632014, India; drmaheshgpillai@gmail.com (M.G.); chinmoykanti.deb@gmail.com (C.K.D.); 5 Department of Soil Science, Swedish University of Agricultural Sciences, Uppsala 750 07, Sweden; 6 Faculty of Fundamentals of Technology, Lublin University of Technology, Nadbystrzycka 38, 20-618 Lublin, Poland; j.szulzyk-cieplak@pollub.pl; 7 Faculty of Environmental Engineering, Lublin University of Technology, Nadbystrzycka 40B, 20-618 Lublin, Poland; g.lagod@pollub.pl

**Keywords:** COD fractionations, molecules size, hydrolysis, OUR, ASM2d, modelling

## Abstract

The activated sludge models (ASMs) commonly used by the International Water Association (IWA) task group are based on chemical oxygen demand (COD) fractionations. However, the proper evaluation of COD fractions, which is crucial for modelling and especially oxygen uptake rate (OUR) predictions, is still under debate. The biodegradation of particulate COD is initiated by the hydrolysis process, which is an integral part of an ASM. This concept has remained in use for over 30 years. The aim of this study was to verify an alternative, more complex, modified (Activated Sludge Model No 2d) ASM2d for modelling the OUR variations and novel procedure for the estimation of a particulate COD fraction through the implementation of the GPS-X software (Hydromantis Environmental Software Solutions, Inc., Hamilton, ON, Canada) in advanced computer simulations. In comparison to the original ASM2d, the modified model more accurately predicted the OUR behavior of real settled wastewater (SWW) samples and SWW after coagulation–flocculation (C–F). The mean absolute relative deviations (MARDs) in OUR were 11.3–29.5% and 18.9–45.8% (original ASM2d) vs. 9.7–15.8% and 11.8–30.3% (modified ASM2d) for the SWW and the C–F samples, respectively. Moreover, the impact of the COD fraction forms and molecules size on the hydrolysis process rate was developed by integrated OUR batch tests in activated sludge modelling.

## 1. Introduction

Nowadays, the European Union (EU) runs programs devoted to the support of research activities that are aimed at developing new environmental technologies. Wastewater treatment is one of the major points of interest in these programs. Regarding wastewater treatment, the effect of a readily biodegradable (S_S_) fraction in activated sludge (AS) has been extensively investigated, but only minor information can be found about the evaluation and behavior of a slowly biodegradable substrate (X_S_) on the oxygen uptake rate (OUR) and chemical oxygen demand (COD) removal in AS systems [1]. The definition of the effects of the effective use of X_S_ during COD removal and OUR may help full-scale wastewater treatment plants (WWTPs) to fulfill the limits that were established in accordance with the EU Directive 91/271 [2]. The effects of X_S_, in the form of particulate and/or colloidal organic substrates, can be important in terms of the COD and nutrient removal processes [3], as well as in the biogas production in anaerobic digesters [4,5]. This is because the X_S_ fraction supplies a significant amount of biodegradable substrates (as a conversion into readily biodegradable material) to the AS process. Knowledge on detailed wastewater characteristics is important in terms of the modelling and optimization of biochemical processes (e.g., the denitrification and enhanced biological phosphorus removal (EBPR)) in modern cost-effective biological nutrient removal (BNR) AS systems.

In the literature, Gori et al. [6] concluded that an increase in the particulate COD that is removed in primary clarifiers would result in a reduction of the energy demand for aeration in the bioreactor, as well as the associated direct carbon dioxide (CO_2_) emissions from microbial respiration and indirect CO_2_ emissions from the power consumption for aeration. However, the same authors, Gori et al. [6], noted that care must be taken during process analysis because a fraction of the COD is necessary for a proper nutrient removal. It is also important to evaluate the kinetics of the biochemical processes that are enhanced by the X_S_ when it is used as an alternative carbon source in full-scale AS systems. The obtained information could be used to optimize the operation of full-scale WWTPs and to provide the guidelines for designing more efficient activated sludge bioreactors. Additionally, other studies regarding the development of wastewater treatment technologies and the optimization of the processes have been carried out and implemented. They have concerned issues related to the development of technologies that use activated sludge in various forms [7,8,9,10,11]; the bioaugmentation of reactors [12,13], biofilm, and hybrid techniques [14,15]; and the preliminary preparation and pre-treatment of sewage sludge and back-side waters [16,17]. New research has also covered alternative technologies, e.g., sorption which may be used for the removal of a big amount of organic and inorganic pollutants and which can be used in conjunction with several techniques (such as flocculation–coagulation) [18,19,20].

The respirometric batch tests constitute a widely accepted method of evaluating the consumption of biodegradable substrates and the OUR [21,22]. An appropriate approach to study OUR is by the means of modelling tools [23]. By using this technique, OUR and COD can be accurately linked in AS systems at the same time that fitted degradation kinetics are obtained.

In this context, the study was divided into two major stages. In the first stage, the examination procedure of X_S_ determination, based on the work of Goel et al. [24], was developed and implemented. In the second stage, a two-steps hydrolysis model, based on that which was previously presented by Orhon et al. [25] and called the “dual-hydrolysis” phenomena, was developed. Finally, the experimental results were used to evaluate the particulate COD fraction impact on OUR modelling according to the two-step hydrolysis process, which was implemented in modified the Activated Sludge Model No 2d (ASM2d) and developed in the previous work [26]. The modified ASM2d model incorporates the rapidly hydrolysable substrate (X_SH_) under the new processes of aerobic, anoxic, and anaerobic hydrolysis according to the concept of two-step hydrolysis. However, the greatest impact was found under aerobic conditions, and a more comprehensive study was conducted that used a respirometric OUR batch test. The aim of this study was to verify an alternative, more complex modified ASM2d for modelling OUR variations as well as a novel procedure for the estimation of the particulate COD fraction by the implementation of the GPS-x platform (Hydromantis, Environmental Software Solutions, Inc., Hamilton, ON, Canada) in advanced computer simulations. The preliminary results of the respirometric batch tests obtained at a large biological nutrient removal (BNR) WWTP in northern Poland, and the modified ASM2d was presented earlier by Drewnowski and Makinia [4,26]. This paper contains further results provided by an experimental database under transient conditions (winter, spring and summer study sessions) for a more comparative study on both (ASM2d and its modification) model predictions while taking the COD fraction forms and molecules size on the hydrolysis process into account.

## 2. Results and Discussion

### 2.1. Wastewater and Biomass Characterization, Including New X_SH_ Fraction

The main characteristics of the SWW at the studied full-scale Wschod WWTP in Gdansk are presented in Table 1. The wastewater was characterized according to Makinia [27] and the Dutch Foundation for Applied Water Research ( STOWA) guidelines [28].

The estimated soluble biodegradable fractions (S_S_), resulting from the sum of soluble, readily biodegradable fermentable organic substrate (S_F_) and soluble, readily biodegradable fermentation products (S_A_) accounted for 19.0–23.9% of total COD. These results were within the range that was previously published in the literature by Makinia [27], where S_S_ accounted for 23.4–28.0% of total COD. Additionally, the presented values by Ekama et al. [29], Henze et al. [30], Kappeler and Gujer [31] and Lesouef et al. [32] for SWW from different WWTPs were similar to the results that are reported in this study. Regarding the inert particulate organic material fraction (X_I_), the estimated X_I_ was within the range of 16.1–28.8%; these results are similar to those that were obtained by Makinia [27]. Moreover, Roeleveld and van Loosdrecht [28] performed comparative studies where the level of X_I_ fraction was reported as 39% of the total COD, but even higher values could be found in the literature. For example, the calibrated X_I_ fraction was accounted by Petersen et al. [33] as up to 50% of total COD. This exceptional situation was explained by the author as a flush effect in the collection systems caused by a rain event. The results obtained in this work were comparable to those found in other influent wastewater characterization referring to the X_S_/X_I_ ratio in the AS systems [34,35].

**Figure 1:**
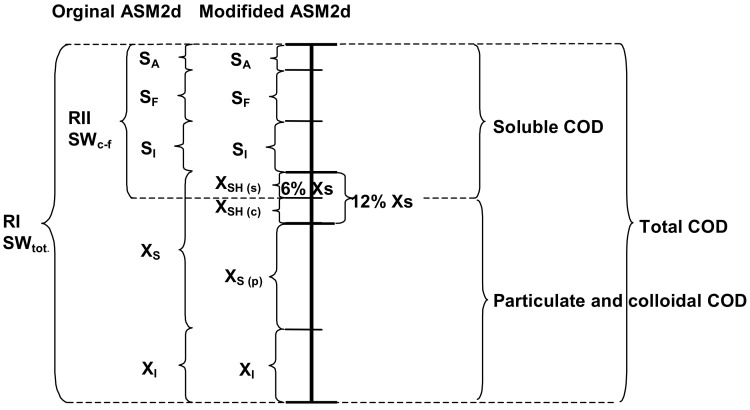
Comparison of the forms and molecules size on COD fractionation evaluated from oxygen uptake rate (OUR) batch tests with the SWW pretreatment and after coagulation–flocculation (C–F) by the modelling hydrolysis process in the original and modified ASM2d (developed according the concept of Melcer et al. [38]).

Once characterized, wastewater constituted a starting point for the ASM2d calibration with the experimental data. The first stage was to use the default stoichiometric and kinetic coefficients, which were defined by Henze et al. [36]. According to the literature [37] and with the aim to minimize the influence of each calibration step on the previously fitted processes, the coefficients were obtained after several iteration loops. In each iteration loop, the steady state results served as a starting point or the subsequent simulation. In regard to the new parameters that were included in the modified ASM2d, a model based on the two-steps hydrolysis was calibrated with the results of the OUR batch tests by using AS and wastewater from the full-scale Wschod WWTP. This approach was employed because the modified ASM2d model involved the new rapidly hydrolysable substrate (X_SH_) component under the three additional processes: the anaerobic, anoxic and aerobic hydrolysis of X_SH_. A scheme of the new fractionation of the wastewater characteristic is presented in Figure 1.

**Figure 2:**
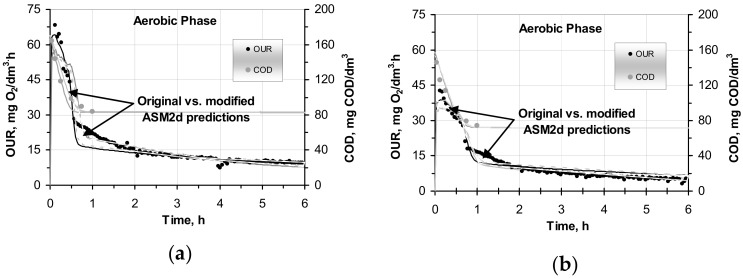
Measured data vs. the original (solid lines) and the modified (shaded lines) ASM2d after optimization step for OUR batch tests in two parallel reactors with mixed liquor from Wschod WWTP: (**a**) the SWW without pretreatment and (**b**) the SWW after C–F.

The results of the modified ASM2d, which was calibrated/validated by using the results of the respirometric batch tests (conventional OUR) from the Wschod WWTP, are presented in Figure 2a,b. The list of the ASM2d default values of the kinetic and stoichiometric parameters and the values that were adjusted during original vs. modified ASM2d calibration were presented earlier by Drewnowski and Makinia [26]. Moreover, the previous study of Makinia and Czerwionka [39] provided the experimental database for the comparison of both model predictions and was used to estimate the amount of X_SH_ (Figure 1 and Table 2). 

In this study, a novel composition of the COD substrate, including the new X_SH_ and three sub-components (particulate, colloidal and soluble X_SH(P/C/S)_ as a part X_S_ was developed in the modified ASM2d according to the COD fractionation concept that was proposed by Melcer et al. [38]. For example, in the OUR batch tests with the SWW without pretreatment, the average value of colloidal/soluble fraction (X_SH (S/C)_ = 12% of X_S_ in the ASM2d) was evaluated from the actual data from the Wschod WWTP by using extended COD substrate calculations (Table 2) and additional OUR batch tests that were integrated with modeling and computer simulation predictions, which were carried out with the GPS-x advanced computer simulation platform (Figure 2). In the SWW sample after C–F, it was necessary to divide an X_SH_ substrate with the new sub-component soluble fraction X_SH (S)_ in order to estimate the new value (6% of X_S_ in the original ASM2d) to fit all the series of the OUR batch tests predictions to the kinetic parameters of the modified ASM2d that was proposed for the SWW without pretreatment. The average composition of the mixed liquor with biomass and wastewater at the modified ASM2d, including the new X_SH_ component, was estimated for both OUR batch tests with the SWW without pretreatment and after C–F during the three (winter, spring and summer) study sessions at the Wschod WWTP (Table 3).

### 2.2. Influence of COD Fractions on Modelling OUR

The research was divided into several stages. After the identification and estimation of the new X_SH_ component, the predictions of OUR and COD removal in the original and the modified ASM2d models were compared. Due to the organization of investigation in this study, the OUR batch tests were carried out in parallel by using the SWW and C–F wastewater samples. As was mentioned before, the X_SH_ fraction was further subdivided into distinct forms: the particulate X_SH,(P)_ and soluble/colloidal X_SH,(S/C)_ in the case of the SWW without pretreatment and only truly soluble X_SH,(S)_ in the case of SWW after C–F. The estimation of the X_SH_ in the SWW without any pretreatment resulted in an average value of 12% from the whole of X_S_ in the modified ASM2d, which was confirmed by mathematical modeling and computer simulation predictions carried out with the GPS-x. The results showed that measured data vs. the original and modified ASM2d after the optimization step for OUR batch tests in two parallel reactors with mixed liquor from Wschod WWTP were as follows: the SWW without pretreatment and the SWW after C–F better predicted the actual changes of the OUR profiles. The sample of experimental results and the fitting that was obtained with both models are presented in Figure 2.

The experimental determination of the kinetic and stoichiometric parameters under aerobic conditions involved the model-based evaluation and curve fitting of OUR profiles in a lab-scale batch test or semi-continuous reactors by using wastewater and heterotrophic biomass [40]. The experimental results were evaluated by using both mathematical modeling and computer simulations to determine the COD fractions in the wastewater, as is presented in Figure 1 and Table 2 and Table 3, as well as an advanced study on two-step hydrolysis process. The stoichiometric and kinetic coefficients (Y_H_, hydrolysis rate (*k*_hyd_), *k*_hyd_,_r_, *K*_X_, and *K*_Xr_) for the two-step hydrolysis were determined by using the simplex method that was developed by Nelder and Mead, and during this study were implemented to the ASM2d. Such an experimental approach has been extensively discussed in the literature [41,42].

The mathematical model was calibrated with one set of data (under summer season) under steady state conditions. The calibrated parameters were within or close to the range of the values presented in the literature [37]. Then, the latter results of the experimental database were gathered with other study data sets (winter and spring seasons) under the same batch tests conditions, with the exceptions of the temperature.

From the fitting, the obtained value for the stoichiometric coefficient Y_H_ was 0.65 (the defaults value in the ASM2d is 0.625). After the mathematical modelling, the best fitting of the kinetic coefficients values for OUR batch tests were *k*_hyd_ = 2.0, *k*_hyd,r_ = 10, and *K*_X_ = 0.1. In the case of parallel samples with the SWW after C–F, it was necessary to divide the X_SH_ component and determine the value of the soluble X_SH,(_s_)_ fraction in order to obtain the best fit between the measured and calculated values in the OUR batch test based on the same kinetic and stoichiometric coefficients, as it was set for the sample of SWW without pretreatment. The average values of X_SH_ and all subdivided components in the particulate, colloidal and soluble forms were optimized by fitted stoichiometric and kinetic parameters. Finally, by using a model developer in the GPS-x platform, the original ASM2d model was extended and modified according to the results of this study in order to compare the experimental OUR tests with the simulations while paying special attention to the hydrolysis process.

### 2.3. Modelling Hydrolysis Process under OUR Batch Tests in the Original and Modified ASM2d

Once the modified ASM2d by Drewnowski and Makinia [26] was calibrated, the models were validated by using a new dataset under seasonal transient (winter and spring) operating conditions, which provided the experimental database for the comparison of both the original and modified ASM2d model predictions.

In comparison with the original ASM2d, the modified ASM2d better predicted the COD and OUR behavior in both cases when fitting with SWW without pretreatment and with wastewater after C–F (see Figure 2a,b). The deviations between the experimental measured data and both models predictions were strongly correlated (r2 = 0.973–0.991) in the parallel reactors that dealt with the SWW without pretreatment and with the wastewater after C–F.

**Figure 3:**
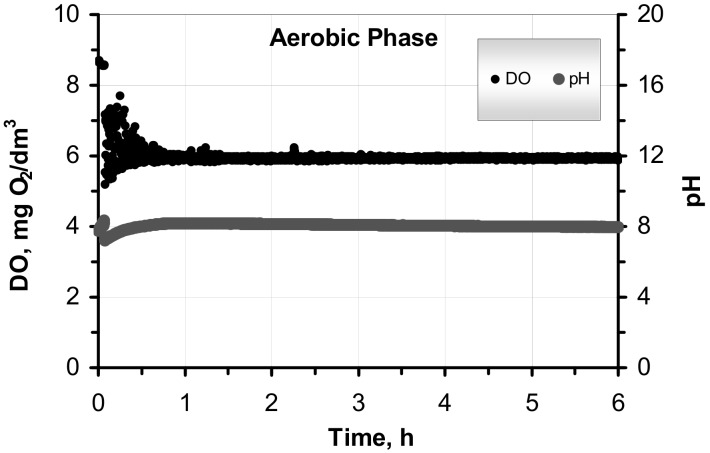
DO and pH on-line profiles that were observed during the OUR tests.

The ASM2d model predictions were re-evaluated according to the further results of the OUR batch tests with the SWW without pretreatment and after C–F under the winter, spring and summer study sessions. The predictive capabilities of the original and modified ASM2d were confirmed by mean absolute relative deviations (MARD) during all study sessions (Table 4). In the comparison with the ASM2d, the modified ASM2d model showed a better prediction ability in the OUR batch tests. The accurate predictions of the modified ASM2d were confirmed by low MARDs, which were in the range of 9.7–15.8% and 11.8–30.3% in the samples with the SWW without pretreatment and after C–F, respectively. For comparison, the corresponding errors that were obtained with the original ASM2d ranged between 11.3–29.5% and 18.9–45.8%. The measured process rates of OURs were better fitted by the modified ASM2d than by the original ASM2d. Additionally, the on-line profile of pH and DO during each of the OUR batch tests were measured and used as real-time control parameters for mathematical modelling (Figure 3). No significant dependence on pH and DO was observed during the OUR tests. A slight pH reduction was observed at the beginning, and this could be explained by the ATU addition at the beginning of aerobic phase, which was aerated to a DO = 6 mg O_2_/dm^3^ set point. The pH value slightly increased at a very slow rate to finally reach pH 8 at the end of the experiment. The accumulated CO_2_ from the respiration activity might have caused the pH to increase at the later part of aerobic phase. Nevertheless, the pH in the bulk wastewater was always maintained in a narrow range of 7–8. However, a strong pH dependency is typical for an enzymatic reaction with a maximum activity around pH 8, as well as a reaction with low activity at pH values below 6 and above 9 [43].

### 2.4. Modelling Hydrolysis Rate vs Settling Velocity of the COD Forms and Particle Size of Molecules

**Figure 4:**
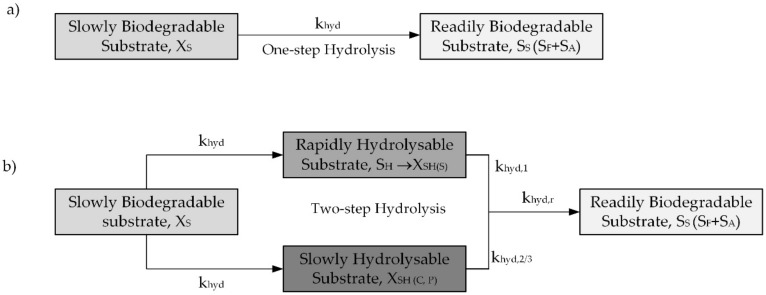
The concept of one/two-steps hydrolysis model based on the original (**a**) vs. the modified (Activated Sludge Model No 2d) ASM2d that was proposed by Drewnowski and Makinia [26] (**b**) and verified by Makinia and Czerwionka [39], as well as by Maruéjouls et al. [47], depending on chemical oxygen demand (COD) forms/molecule size. Note: More details regarding the two-step hydrolysis might be found in Table S1 in Supplementary Materials.

The study was focused more specifically on the variation of the hydrolysis rates due to the physical properties of particulate COD such as X_S_ and its influence on the hydrolysis rate in the activated sludge models (ASMs). The research showed that increasing the particle size decreased biodegradability. Similar results have been reported in other studies [43,44,45,46]. Unfortunately, the mentioned phenomenon has not been not well accounted for in the ASM models, including the one employed in this study. Because of that, the original ASM2d model should be incorporated X_S_ into the rapidly hydrolysable substrate (X_SH_) under the processes of aerobic, anoxic, and anaerobic hydrolysis, according to the concept of two-step hydrolysis (Figure 4). Moreover, in advanced activated sludge systems, a variation of the hydrolysis rates may have a significant influence on the readily biodegradable organic matter (S_S_) that is available for denitrification in the course of hydrolysis in the anoxic zone. Low hydrolysis rates slow down the hydrolysis of particulate matter (e.g., X_S_); thus, S_S_ will be released in the aerobic reactors rather than in the first anoxic activated sludge reactor. Therefore, S_S_ will be subjected to aerobic removal during nitrification, consuming a greater amount of energy and oxygen. A greater sludge mass may occur in the system as a result of longer particulate matter presence; this, in turn, leads to an increased sludge load on the secondary clarifiers. According to Maruéjouls et al. [47], nitrification may become endangered if sludge wastage has to be decreased.

In this paper, an improved model of hydrolysis that involved a modified ASM2d and that took the particle forms (soluble, colloidal, particulate) of COD properties (such small, medium, and large molecules) into account was employed. As far as theoretical hydrolysis models are not fully capable of predicting field observations, different models with various levels of complexity have been proposed in the literature. The hydrolysis mechanisms are well-known: If a molecule of COD substrate is too large and cannot be assimilated and degraded by biomass, it needs to undergo hydrolysis. Research, such as [48], has reported two concepts of hydrolysis: 1) The enzymes that are produced by bacteria are released and adsorbed on the surface of a particle, and 2) bacteria are attached on the particle surface, so enzymes are secreted and a soluble substrate is thus produced. In either concept, hydrolysis constitutes a process that is surface site-limited. However, it is still not known to what extent the chemical and physical properties of a particle influence the rates of hydrolysis and kinetics.

Morgenroth et al. [46] described different hydrolysis kinetics while reviewing hydrolysis modeling in the treatment of aerobic wastewater. The stoichiometry of a reaction can be altered by the differentiation of the bacterial populations that are capable of degrading the soluble or particulate substrates. The rates of hydrolysis are governed by the kind of organic particles (slow, medium or fast), and these rates are also included in recent models. Hydrolysis can be sequential or parallel; in the former, the slowly hydrolysable substrate X_S_ is transformed into medium hydrolysis rate X_S_ and finally into X_SH_, which is readily hydrolysable molecule form [49]. In the latter, all organic particles are directly hydrolyzed into a soluble substrate, albeit at different rates [41,45]. Two surface-based hydrolysis models for the activated sludge were employed by Dimock and Morgenroth [50]. In the so-called shrinking particle model, the diameter of a particle is continuously decreased in the course of hydrolysis. In turn, the second one, i.e., particle breakup model, assumes that the breakup of particles contributes to an increase in the surface area of particles. Data collected from the WWTP influent, with different COD fractions that are determined with specific rates of hydrolysis, are used for the calibration of these models. The data are employed in order to obtain a fixed COD fractionation that is independent of the changes in the wastewater composition that are caused by diverse upstream conditions (including biodegradation, settling or resuspension in sewers, and wet/dry weather), as was presented by Maruéjouls et al. [47].

The goal of the presented research was to show the potential of these particulate forms of COD in order to better describe the hydrolysis that occurs in the course of the wastewater treatment bioprocesses (e.g., pre-denitrification and denitrification–nitrification) in an activated sludge system and to better predict the quality of the WWTP effluent. This paper compared the calibration and validation of the original and modified ASM2d models by using experimental data that were obtained by conducting respirometric OUR tests on wastewater samples that were fractionated by different sizes of COD molecules and their forms, as well as from studies conducted by the authors [26,51,52,53,54,55]. For example, the experiments that were performed in [56] showed that when wastewater flows from an urban catchment to the biological treatment, the particle settling velocity distribution (PSVD) becomes highly differentiated. Moreover, this study showed that the model enables the reproduction of the dynamic PSVD evolution of wastewater while the suspended solids flow through the integrated urban wastewater system, which comprises an urban catchment, a combined sewer, retention tanks, a grit chamber, and a primary clarifier under dry and wet weather conditions [57,58]. Maruéjouls et al. [47] also discussed an original concept that linked PSVD with hydrolysis rates, as well as the influence of ASM model parameters on the quality of the effluent.

**Figure 5:**
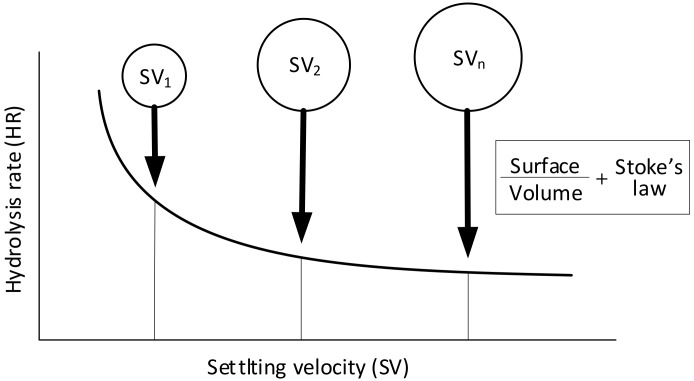
The hydrolysis rate constants in regard to the particle settling velocity (SV) according to the model that was proposed by Maruéjouls et al. [52].

For example, in the ASM1, the slowly biodegradable organic substrate (X_S_) and slowly biodegradable organic nitrogen (X_ND_) undergo hydrolysis in readily biodegradable matter (S_S_) and readily biodegradable organic nitrogen (S_ND_), respectively. The approach that is used in the PSVD model is almost identical; the only difference is that X_S_ and X_ND_ are fractionated in several particle classes, enabling the setting-up of different hydrolysis rates, depending on the particle sizes, that are approximated by the settling velocity. In the WWTP model, the X_S_ and the organic nitrogen products that are obtained in the course of biomass decay are evenly distributed among the first five classes with the highest rates of hydrolysis, because it is assumed that the lower hydrolysis rates are relevant for the particles that are characterized by high settling velocities—which are almost entirely removed in primary clarifiers, as reported by Maruéjouls et al. [47]. Therefore, in the presented research, a novel modeling approach that used a modified ASM2d and that pertained to the impact of the COD fraction forms and particle sizes of molecules on the process of hydrolysis was devised by comparing the OUR tests conducted with the activated sludge biomass from an integrated urban WWTP system. This approach drew on the concept, suggested Maruéjouls et al. [47], that PSVD is a determining factor for the prediction of the wastewater quality along a catchment, a combined sewer, or a primary treatment system. This research, as well as studies conducted by other authors [47,51,52,53,54,55,57], confirms that a comparative study of the original and modified ASM2d models that accounts the effects of the COD fraction and particles sizes of molecules on the hydrolysis process should be applied under transient conditions (summer, spring and winter). The distribution data were fed into the hydrolysis model that involved a comparison of results on the wastewater quality that were obtained in the course of simulations that were performed at the hydrolysis rates of particles (such as molecules size and forms of COD substrates) that depended on the OUR batch tests as well as the conditions of the settling velocities (Figure 5). It was indicated that at a classic constant rate of hydrolysis, the concentration of nitrogen in the WWTP effluent was mostly overestimated during the pollutant concentration peaks, though this was found to have also been caused by wet/dry weather or seasonal (spring, summer, fall, winter) changes in the year. The internal carbon source, e.g., the transition of the substrates such as from the X_S_ to S_S_ molecular form, in the single-step hydrolysis model in the original ASM2d, as well as the oxygen demand, can vary by 30–50% in the first aerated tank within a pre-denitrifying AS system. The obtained results indicate the relevance of considering PSVD and its influence on the rates of hydrolysis for predicting the quality of the WWTP effluent by means of the original and modified ASM2d, where the sequential two-step hydrolysis concept and was first implemented by Drewnowski and Makinia [26]. The dynamics of numerous processes along the wastewater line, including the settling that occurs in various wastewater subsystems, including retention tanks, grit chambers, primary and secondary settlers, sewers and rivers, are governed by such characteristics as PSVD, X_S_, and the particle size of COD forms. In other investigations, e.g., that of Maruéjouls et al. [47], it has also been suggested that biological treatment processes, including biofilters, biofilms, activated sludge systems, or anaerobic digestion, can be significantly influenced by particle size and settling velocity (SV).

This kinetics yields the hydrolysis rate (k_hyd_), which is hyperbolically dependent upon the particle radius (see Equation (1)):

(1) $$k_{\mathrm{hyd}}\;=\;\omega \cdot \frac{\mathrm{Sphere}\;\mathrm{surface}}{\mathrm{Sphere}\;\mathrm{volume}}\;=\;\omega \cdot \frac{3}{\mathrm{radius}}$$

Moreover, in accordance with Stokes’ law, the settling velocity is a function of the squared radius (Equation (2)):

(2) $$\mathrm{radius}\;=\;f\left(\sqrt{V_{S}}\right)$$

By replacing the radius by a function of *V*_S_:

(3) $$k_{\mathrm{hyd}(1,2,3\ldots n)}\;=\;\alpha \cdot \frac{1}{\sqrt{V_{S(1,2,3\ldots n)}}}$$

Subsequently, Equation (3) enables the calculation of the hydrolysis rates (*k*_hyd 1,2,3…n_) for various particle settling velocities (*SV*_1,2,3..n_). In the aforementioned equations, *k*_hyd(1,2,3…n)_ corresponds to the different hydrolysis rates (gCOD/(gCOD/d)) that depend on molecules size and form—which could give the *k*_hyd,r_, which is the average surface specific hydrolysis rate constant (gCOD/m/(gCOD/d))—and α denotes a constant coefficient.

To the best of the authors’ knowledge, there are only a few models that link the particle size of COD molecules to the hydrolysis rate, e.g., [50]. Nevertheless, no thorough study that links the particle settling velocity to the hydrolysis rate has been performed thus far. Therefore, a conceptual model was proposed in the study in order to extend the modified ASM2d that was proposed by Drewnowski and Makinia [26]. On the basis of concept that was presented by Maruéjouls et al. [47], this study also confirms that hydrolysis constitutes a surface-limited reaction in the biochemical processes of activated sludge; this study also confirms that hydrolysis rates decrease along with the particle radius and, in line with Stokes’ law, with particle settling velocity, as presented in Equations (1)–(3) and Figure 5.

## 3. Materials and Methods

### 3.1. Wastewater and Biomass Characterization for Lab Tests and Modelling

During the study at the Wschod full-scale WWTP, Poland, the experiments were performed at process temperatures (12–21 °C) according to the real conditions in full-scale bioreactors. These bioreactors were run in the Modified University of Cape Town (MUCT) process configuration. More details from the Wschod WWTP can be found elsewhere [25]. For the laboratory OUR batch tests, the mixed liquor and settled wastewater (SWW) samples were collected from the studied full-scale WWTP during winter, spring and summer study periods. For the wastewater characterization, the average daily, every 1/24-h time-proportional samples of the settled wastewater (after the first stage of mechanical treatment) were collected. The wastewater characterization is presented in Table 5.

### 3.2. Conventional OUR Batch Test Measurement

**Figure 6:**
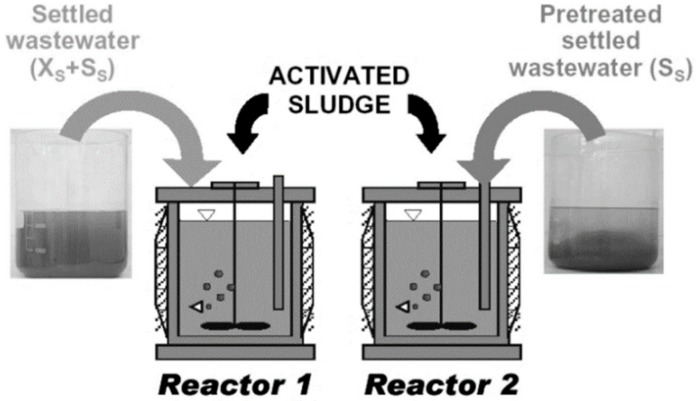
The experimental procedure with two parallel batch reactors (developed by Drewnowski and Makinia [59]).

According to the procedure that was previously described in the literature by Drewnowski and Makinia [59], the laboratory batch-tests were conducted in parallel reactors. For example, the aerobic laboratory OUR batch tests were carried out with SWW without any pretreatment and with the same portion of wastewater, but they were pretreated with the coagulation–flocculation (C–F) method, as presented in Figure 6. The physical–chemical (C–F) procedure was adapted from a paper by Mamais et al. [60] in order to achieve only a truly soluble organic fraction in wastewater for a comprehensive study on the particulate COD fraction impact on OUR modelling in AS. In each period (winter, spring and summer), batch tests were carried out in duplicate for the OUR measurements. Before starting the OUR experiments, 10 mg/dm^3^ of allylthiourea (ATU), a nitrification inhibitor, was added to inhibit oxygen consumption due to the nitrification process. After placing the mixed liquor in the reactors, the dissolved oxygen (DO) set point was kept at 6 g O_2_/m^3^, and the automated OUR measurements were initiated. The filtered mixed liquor samples were analyzed for COD. Additionally, aerobic experiments were carried out to determine the growth yield coefficient, Y_H_ On the basis of the OUR measurements and COD removal, the Y_H_ coefficient and the kinetics of the hydrolysis process were determined according to the procedure that was previously described in the literature [29,31].

### 3.3. Organization of the Modelling Study

The models were calibrated and validated by using the experimental results from the OUR batch respirometric tests and from measurements in the full-scale MUCT bioreactor. The results from the parallel batch experiments carried out with the wastewater samples of SWW without pretreatment and after C–F were used with the original and modified ASM2d to compare the predictions of both models in terms of the COD and OUR behavior. Mathematical modelling was carried out with the GPS-x advanced computer simulation platform (Hydromantis Environmental Software Solutions, Inc., Hamilton, ON, Canada), and an additional model developer tool was used in order to create the modified ASM2d model as well as to implement the two-step hydrolysis concept [61]. The stoichiometric and kinetic parameters were numerically optimized by using the Nelder–Mead simplex method [62]. The modified ASM2d incorporated an X_SH_ component under new processes according to the concept of the two-step hydrolysis that is presented in Figure 4. A previous study regarding the two-step hydrolysis was also done by Drewnowski and Makinia [26].

### 3.4. Analytical Methods

A Hach test-in-tube Xion 500 spectrophotometer (Hach Lange GmbH, Dusseldorf Germany) was used to determine the NH_4_-N, NO_3_-N, PO_4_-P and total/soluble COD, whereas a Total Organic Carbon (TOC)/Total Nitrogen (TN) nalyzer (SHIMADZU Corporation, Kyoto, Japan) was used to measure the TN concentration. The analytical procedures that were adapted by Hach Lange GmbH (Germany) were followed as suggested by American Public Health Association (APHA) [63]. The Polish Standards (PN-72/C-04559) were followed in terms of the total suspended solids (TSS) and volatile suspended solids (VSS). The WTW Measurement Systems, Inc. (WTW) CellOx^®^ 325 DO sensor with integrated temperature compensation and membrane leak monitoring was used to measure the DO concentration and keep the DO at set point in the laboratory reactors during the batch tests. Additionally, a WTW SitrrOx G DO probe was used to measure the actual OUR, as its features included a quick response time (t99 < 60 s) and low-maintenance operation. Moreover, several parameters (including DO, pH, temperature and oxidation reduction potential (ORP)) were determined and recorded by using the WTW probes that were installed in the batch reactor and connected to the computer.

## 4. Conclusions

A complex model that takes many different processes into account needs the calibration and validation of a large number of data and model parameters, as well as the initial concentrations of model components. Naturally, there is no model that is perfectly situated for all cases; such variables vary and hence need to be determined for every model application. Unfortunately, this is often faced with practical engineering problems.

Therefore, this study showed that the mechanism of the hydrolysis process when using the ASM2d is not adequate to predict the aerobic behavior of OUR in batch experiments, and the two-steps hydrolysis model, implemented in a modified ASM2d, is more flexible and might be recommended for modelling the respirometric batch test due to the better fit to OUR variations. Moreover, from the results obtained in this work, it could be concluded that:

1. OUR measurements, together with COD consumption, are a versatile tool for characterizing the biodegradability of organic matter and could be used for evaluations of conceptual models as well as the modelling and optimization of biochemical processes (e.g., denitrification and EBPR) in modern, cost-effective BNR-activated sludge systems.
2. A new approach of particulate COD substrate modelling involving the use of OUR in activated sludge systems is necessary for adequate hydrolysis model prediction and could be recommended for monitoring the wastewater and activated sludge conditions.
3. Hydrolysis constitutes a surface-limited reaction in the biochemical processes of activated sludge, and hydrolysis rates decrease along with the particle radius and, in line with Stokes’ law, with particle settling velocity.
4. The definition of a new slowly biodegradable component (the X_SH_ fraction was subdivided into distinct forms: the particulate, colloidal and soluble) and three new processes enhanced the OUR description of the activated sludge processes in the ASM2d.
5. In comparison with the original ASM2d, the modified ASM2d model more accurately predicted the aerobic behavior of OUR. The deviations between both models with respect to the above-mentioned predictions were strongly correlated (r^2^ = 0.973–0.991) in the parallel reactors that dealt with the SWW without pretreatment and with the wastewater after C–F.
6. The average MARDs were 11.3–29.5% and 18.9–45.8% (original ASM2d) vs. 9.7–15.8% and 11.8–30.3% (modified ASM2d) in the samples with the SWW without pretreatment and after C–F, respectively.
7. In contrast, the MARD differences of COD concentrations, which were measured during the OUR tests, were in a narrow range (0.15–1.9%) for both the models with the SWW without pretreatment and those after C–F.
8. Simulations in the GPS-X platform version from 6.5 to 7.0 showed that operating conditions in which the predictions were better appeared when the technological system lacked a primary settling tank—this is the case of typical WWTPs functioning as sequential batch reactors with activated sludge.

## Figures and Tables

**Table 1:** Sample results of the COD fractions in the settled wastewater (SWW) at the studied full-scale Wschod WWTP.

Wschod WWTP	COD Fraction	Calculated Values During the Study Period					
		Winter		Spring		Summer	
		Concentration COD		Concentration COD		Concentration COD	
		[g COD/m^3^]	[%]	[g COD/m^3^]	[%]	[g COD/m^3^]	[%]
**Settled wastewater fractionation**	S_I_	38.9	6.2	37.1	5.4	36.1	4.5
	S_F_	64.1	10.3	66.1	9.6	64.9	8.2
	S_A_	85.0	13.6	87.7	12.7	86.0	10.8
	X_I_	133.5	21.4	181.3	26.2	257.4	32.4
	X_S_	302.5	48.5	318.8	46.1	349.6	44.1
	Total COD	624.0	100.0	691.0	100.0	794.0	100.0

**Table 2:** Estimation of the rapidly hydrolysable substrate (X_SH_) component in the modified ASM2d based on the results of this study and previous investigations that were reported by Makinia and Czerwionka [39].

Series	Wschod WWTP				
	Settled Wastewater			Colloidal Fraction	Estimation of X_SH (S/C)_
	COD_inf_	COD_f(1.2),inf_	COD_f(0.1),inf_	COD_col,inf_	% of SBCOD
	[g COD/m^3^]			[g COD/m^3^]	
1	650	237	213	24	7.5
2	630	188	152	36	11.8
3	590	182	143	39	13.6
4	548	164	131	33	12.3
5	451	138	106	32	14.5
6	821	191	154	37	9.2
7	620	176	132	44	14.5
8	1390	247	174	73	10.7
9	1001	236	172	64	13.0
10	797	165	112	53	13.6
Ave. value	750	192	149	44	12

Note: COD_inf_—grab sample; inflow of total COD in settled wastewater; COD_col,inf_—grab sample; inflow of colloidal COD in settled wastewater; COD_f(1.2),inf_—grab sample; soluble COD analysis after filtration (GF/C = 1.2 μm); COD_f(0.1),inf_—grab sample; soluble COD analysis after filtration (GF/C = 0.1 μm); and % of SBCOD—% of soluble/colloidal form of X_SH (S/C)_ fraction used in the modified ASM2d.Values were calculated on the average value of X_S_ based on study on COD fractionation in the settled wastewater.

**Table 3:** The average composition of the mixed liquor with biomass and wastewater, including the new X_SH_ component as a part of the slowly biodegradable substrate (X_S_) that was used in the modified ASM2d under seasonal transient operating conditions.

Model	Study Period	Type of Sample	Contribution of the COD Particulate Component [%]										Total
			X_inog_	X_PP_	X_PHA_	X_STO_	X_PAO_	X_A_	X_H_	X_I_	X_SH_	X_S_	%
**Modified ASM2d**	**Winter**	SW	12.0	3.4	0.7	0.0	12.9	1.3	19.6	6.1	0.8	43.2	100.0
		SW c–f	12.8	4.3	0.8	0.0	12.6	1.2	18.9	1.7	0.5	47.2	100.0
	**Spring**	SW	8.3	2.4	0.6	0.0	13.7	0.8	23.4	9.2	1.3	40.3	100.0
		SW c–f	10.2	3.2	0.7	0.0	14.3	0.8	21.4	2.5	0.7	46.2	100.0
	**Summer**	SW	12.3	3.1	0.6	0.0	9.8	1.0	18.8	7.3	1.0	46.1	100.0
		SW c–f	13.1	3.5	0.6	0.0	10.4	1.1	17.5	2.9	0.5	50.3	100.0

**Table 4:** The mean absolute relative deviations (MARD) values for model predictions of COD and OUR process rates in the OUR batch tests that were carried out with the settled wastewater without pretreatment and after C–F from Wschod WWTP.

Experiment	Study Period	Process Rate	MARD [%]			
			Wschod WWTP			
			Settled Wastewater		Settled Wastewater (C–F)	
			ASM2d	Modified ASM2d	ASM2d	Modified ASM2d
**OUR batch test**	Winter	Oxygen uptake	29.5	14.6	45.8	30.3
		COD utilization	4.3	3.8	5.0	3.1
	Summer	Oxygen uptake	11.3	9.7	18.9	11.8
		COD utilization	4.8	5.5	5.2	5.4
	Spring	Oxygen uptake	16.2	15.8	23.3	19.2
		COD utilization	4.3	2.7	4.4	4.5

**Table 5:** Average characteristics of the studied Wschod wastewater treatment plants (WWTP) in Gdansk during the period of the study.

Parameter	PE	Q [m^3^/d]	SRT [d]	MLSS [kg/m^3^]	COD [g COD/m^3^]	SCOD [g COD/m^3^]	TP [g P/m^3^)]	TN [g N/m^3^]	N-NH_4_ [g N/m^3^]	N-NO_3_ [g N/m^3^]
Influent	574,000	81,600	x	x	626 ± 82	194 ± 38	14.9 ± 2.6	81.2 ± 5.0	58.9 ± 3.4	7.4 ± 0.64
Effluent					48 ± 4.2		0.60 ± 0.1	11.1 ± 1.1	1.20 ± 0.75	
Reactor	T = 11.8–20.5 °C		21 ± 2.9	5.45 ± 0.56	x		x	x	x	x

## Supplementary Materials

The following are available online, Table S1: Stoichiometric matrix and process rates for the modified ASM2d model including the new variable X_SH_ and three hydrolysis processes under aerobic, anoxic and aerobic conditions; list of most important abbreviations and symbols.
