# Supplementary material for: The Influence of COD Fraction Forms and Molecules Size on Hydrolysis Process Developed by Comparative OUR Studies in Activated Sludge Modelling
Source: Molecules. 2020 Feb 19;25(4):929. doi: 10.3390/molecules25040929 (PMC7070828; doi:10.3390/molecules25040929)
Supplement: Supplementary file 1 [file molecules-25-00929-s001.pdf]

Supplement A

In the IWA models, e.g. original ASM2d, the conversion from X_S to the hydrolysis product (S_S) is straightforward, because one COD unit of X_S is hydrolyzed into one COD unit of S_S (Fig. 2a and 3). However, the conversion from X_S consumed to S_S is not as readily performed by hydrolysis processes The reason is that hydrolysis of X_S may be insufficient to supply the S_S necessary for the maintenance energy requirement of the biomass under aerobic, anoxic or anaerobic conditions. Analyzing data of this study it can be seen that the S_S produced from hydrolysis of X_S typically can supply most of the S_S needed for maintenance – but not all. A fraction of the S_S produced by hydrolysis of X_S to X_{SH} should be used for biomass maintenance and not for biomass growth. Therefore the modified ASM2d including two different hydrolysis rate (k_{hyd} = 2 and $k_{hydr,r}$ = 10) the new variable X_{SH} and three new hydrolysis processes under aerobic, anoxic and anaerobic conditions as well as three new forms such as soluble, colloidal, particulate of $X_{SH\ (S,C,P)}$ depend on the molecules size vs. settling velocity and corresponds to the average value of specific hydrolysis rate constant, $k_{hyd,r}$ (Fig. 2b and 3).

Table S1. Stoichiometric matrix and process rates for the modified ASM2d model including the new variable X_{SH} and three hydrolysis processes under aerobic, anoxic and aerobic conditions

Process \ Variable	S_F	S_{NH4}	S_{PO4}	S_I	S_{ALK}	X_{SH}	X_S
Aerobic hydrolysis of X_S		$V_{1,NH4}$	$V_{1,PH4}$		$V_{1,ALK}$	1	-1
Anoxic hydrolysis of X_S		$V_{2,NH4}$	$V_{2,PH4}$		$V_{2\ ALK}$	1	-1
Anaerobic hydrolysis of X_S		$V_{3,NH4}$	$V_{3,PH4}$		$V_{3,\ ALK}$	1	-1
Aerobic hydrolysis of $X_{SH\ (S,C,P)}$	$1-f_{SI}$	$V_{22,NH4}$	$V_{22,PH4}$	f_{SI}	$V_{22,ALK}$	-1	
Anoxic hydrolysis of $X_{SH\ (S,C,P)}$	$1-f_{SI}$	$V_{23,NH4}$	$V_{23,PH4}$	f_{SI}	$V_{23,ALK}$	-1	
Anaerobic hydrolysis of $X_{SH\ (S,C,P)}$	$1-f_{SI}$	$V_{24,NH4}$	$V_{24,PH4}$	f_{SI}	$V_{24,ALK}$	-1	

Process	Process rate, ρ_i
Aerobic hydrolysis of X_S	$k_{hyd}\ \frac{S_{O2}}{K_{O2}+S_{O2}}\ \frac{X_S/X_H}{K_X+X_S/X_H}\ X_H$
Anoxic hydrolysis of X_S	$k_{hyd}\ \eta_{NO3}\ \frac{K_{O2}}{K_{O2}+S_{O2}}\ \frac{X_S/X_H}{K_X+X_S/X_H}\ X_H$
Anaerobic hydrolysis of X_S	$k_{hyd}\ \eta_{fe}\ \frac{K_{O2}}{K_{O2}+S_{O2}}\ \frac{K_{NO3}}{K_{NO3}+S_{NO3}}\ \frac{X_S/X_H}{K_X+X_S/X_H}\ X_H$
Aerobic hydrolysis of $X_{SH\ (S,C,P)}$	$k_{hyd,r}\ \frac{S_{O2}}{K_{O2}+S_{O2}}\ \frac{X_{SH(S,C,P)}/X_H}{K_{Xr}+X_{SH(S,C,P)}/X_H}\ X_H$
Anoxic hydrolysis of $X_{SH\ (S,C,P)}$	$k_{hyd,r}\ \eta_{NO3}\ \frac{K_{O2}}{K_{O2}+S_{O2}}\ \frac{X_{SH(S,C,P)}/X_H}{K_{Xr}+X_{SH(S,C,P)}/X_H}\ X_H$
Anaerobic hydrolysis of $X_{SH\ (S,C,P)}$	$k_{hyd,r}\ \eta_{fe}\ \frac{K_{O2}}{K_{O2}+S_{O2}}\ \frac{K_{NO3}}{K_{NO3}+S_{NO3}}\ \frac{X_{SH(S,C,P)}/X_H}{K_{Xr}+X_{SH(S,C,P)}/X_H}\ X_H$

Note: $X_{SH\ (S,C,P)}$ - Slowly Hydrolysable Substrate in different forms such as soluble, colloidal, particulate
S_H - Rapidly Hydrolysable Substrate directly converted from $X_{SH\ (S)}$ – part of X_{SH} (6%) in soluble form
k_{hyd}- Specific Hydrolysis Rate Constant, 1/d
$k_{hyd1,2,3...n}$ - The value of Specific Hydrolysis Rate Constant depend on the molecules size and forms such as soluble, colloidal, particulate for various settling velocities proposed by Drewnowski and Makinia [26] and verified by Makinia and Czerwionka [44] / Maruéjouls et al. [52].
$k_{hyd,r}$- Average Specific Hydrolysis Rate Constant

List of most important abbreviations and symbols:

K_{hyd} – Specific hydrolysis rate constant, 1/d
$K_{NO3,hyd}$ – Nitrate saturation/inhibition coefficient for hydrolysis of slowly biodegradable fraction ,mg N/dm^3
$K_{O2,hyd}$ –Oxygen saturation/inhibition coefficient for hydrolysis of slowly biodegradable fraction,mg O_2/dm^3
K_X – Saturation coefficient for hydrolysis of particulate COD, mg COD/mg COD
OUR – Oxygen uptake rate, mg O_2/g VSS·h
PAO – Phosphate accumulating organism
PHA – Poly-hydroxy-alkanoates
RBCOD – Readily biodegradable COD, mg COD/dm^3
SBR – Sequencing Batch Reactor
SBCOD – Slowly biodegradable COD, mg COD/dm^3
SCOD – Soluble COD, mg COD/dm^3
S_A – Concentration of soluble, readily biodegradable fermentation products, mg COD/dm^3
S_{ALK} – Concentration of alkalinity of the wastewater, mol HCO_3^-/dm^3
S_F – Concentration of soluble, readily biodegradable fermentable organic substrate, mg COD/dm^3
S_I – Concentration of soluble inert organic material, mg COD/dm^3
S_{NH4} – Concentration of ammonium plus ammonia nitrogen, mg N/dm^3
S_{O2} – Concentration of dissolved oxygen, mg O_2/dm^3
S_{PO4} – Concentration of orthophosphate, mg P/dm^3
S_S – Concentration of soluble, readily biodegradable organic substrate, mg COD/dm^3
X_{AUT} – Concentration of autotrophic organisms, mg COD/dm^3
X_H – Concentration of heterotrophic organisms, mg COD/dm^3
X_I – Concentration of inert particulate organic material, mg COD/dm^3
X_S – Concentration of slowly biodegradable substrates, mg COD/dm^3
X_{SH} – Concentration of rapidly hydrolysable substrate, mg COD/dm3
Y_A – Growth yield coefficient for autotrophic organisms, mg COD/mg N
Y_H – Growth yield coefficient for heterotrophic organisms, mg COD/mg COD
η_{fe} – Anaerobic hydrolysis reduction factor, -
$\eta_{NO3,hyd}$ – Anoxic hydrolysis reduction factor, -
